# Preoperative C-reactive protein and other inflammatory markers as predictors of postoperative complications in patients with colorectal neoplasia

**DOI:** 10.1186/s12957-021-02142-4

**Published:** 2021-03-13

**Authors:** Sufana H. Alsaif, Ailín C. Rogers, Priscilla Pua, Paul T. Casey, Geoff G. Aherne, Ann E. Brannigan, Jurgen J. Mulsow, Conor J. Shields, Ronan A. Cahill

**Affiliations:** 1 University College Dublin School of Medicine, Belfield, Dublin 4, Ireland; 2 Department of Surgery, Mater Misericordiae University Hospital (MMUH), 46-47 Eccles Street, Dublin 7, D07 A8NN Ireland; 3 Section of Surgery and Surgical Specialties, School of Medicine, University College Dublin, Dublin, Ireland

**Keywords:** Colorectal cancer, Inflammatory markers, C-reactive protein, Postoperative complications, Clavien-Dindo

## Abstract

**Background:**

Inflammatory markers are measured following colorectal surgery to detect postoperative complications. However, the association of these markers preoperatively with subsequent postoperative course has not yet been usefully studied.

**Aim:**

The aim of this study is to assess the ability of preoperative C-reactive protein (CRP) and other inflammatory marker measurements in the prediction of postoperative morbidity after elective colorectal surgery.

**Methods:**

This is a retrospective study which catalogs 218 patients undergoing elective, potentially curative surgery for colorectal neoplasia. Preoperative laboratory results of the full blood count (FBC), C-reactive protein (CRP) and carcinoembryonic antigen (CEA) were recorded. Multivariable analysis was performed to examine preoperative variables against 30-day postoperative complications by type and grade (Clavien-Dindo (CD)), adjusting for age, sex, BMI, smoking status, medical history, open versus laparoscopic operation, and tumor characteristics.

**Results:**

Elevated preoperative CRP (≥ 5 mg/L) was significantly predictive of all-cause mortality, with an OR of 17.0 (*p* < 0.001) and was the strongest factor to predict a CD morbidity grade ≥ 3 (OR 41.9, *p* < 0.001). Other factors predictive of CD morbidity grade ≥ 3 included smoking, elevated preoperative platelet count and elevated preoperative neutrophil-lymphocyte ratio (OR 15.6, 8.6, and 6.3 respectively, all *p* < 0.05). CRP values above 5.5 mg/L were indicative of all-cause morbidity (AUC = 0.871), and values above 17.5 mg/L predicted severe complications (AUC = 0.934).

**Conclusions:**

Elevated preoperative CRP predicts increased postoperative morbidity in this patient cohort. The results herein aid risk and resource stratification and encourage preoperative assessment of inflammatory propensity besides simple sepsis exclusion.

## Introduction

Inflammatory markers are routinely measured in patients following colorectal surgery to monitor progress alongside clinical parameters [1, 2]. Postoperative C-reactive protein (CRP) levels are particularly useful for the detection of significant postoperative complications [3–5]. While some physiological derangement is expected following operative intervention, absolute levels of inflammatory markers above expected thresholds, prolonged elevations or a second rise after an initial decline all encourage further investigation or intervention [6–8]. This allows for early identification and correction of potential complications, or confident discharge of those with uncomplicated clinical courses.

Levels of inflammatory markers may be elevated before surgery in the presence of an infection, morbidities such as atherosclerosis, or inactivity in a patient presenting for planned operation [9–11]. An elevation in full blood cell (FBC) count, its constituent neutrophil, lymphocyte, and platelet counts, and CRP is also induced by colorectal neoplasia itself, especially in the context of its metabolic consequences which include sarcopenia and cachexia [12]. The importance of systemic mediators in oncological outcome is underlined by the modified Glasgow Prognostic Score (mGPS), which relates high CRP (> 10 mg/L) to cancer recurrence and reduced 5-year survival [13]. It may be the case that systemic inflammation predisposes patients to immunological dysfunction with short and intermediate infectious, inflammatory, and oncological consequences. Patients in a pre-existing pro-inflammatory state are primed for an augmented inflammatory response in the event of additional provocation such as surgery [14]. Despite the presence of considerable data regarding inflammatory mediation, little study to date has examined the propensity for preoperative inflammatory markers to relate to early postoperative complications, with most work instead being presently directed towards their use as snapshot indicators rather than as reflectors of biological balance throughout the perioperative period [15–19].

This clinical study was performed to test the hypothesis that elevated preoperative inflammatory markers, particularly CRP, in patients without overt sepsis, may signify a complicated early postoperative course following elective colorectal resection. Such markers may thereby act as early risk stratification tools for patients potentially facing clinical deviation from expected trajectories.

## Methods

### Inclusion and exclusion criteria

Patients who underwent elective, potentially curative resection for colorectal neoplasia in our institution, the Mater Misericordiae University Hospital, between January 2009 and December 2016 were included for retrospective analysis. Cases of dysplastic tumors were also included and adjusted for in the analysis. Patients with second cancers, both synchronous and metachronous, and inflammatory bowel disease and those that underwent neoadjuvant chemotherapy or radiation therapy were excluded from the study, in addition to those admitted with fever or sepsis, defined as two or more of the Systemic Inflammatory Response Syndrome criteria plus a microbial source that is either suspected or confirmed with blood cultures [19]. Patients without any laboratory data or postoperative outcome documentation were excluded from analysis, while those with incomplete datasets were included in the relevant subcategory analysis only.

### Patient management

Patients planned for elective surgery in our institution routinely complete a full diagnostic work-up, which includes computerized tomography of the chest, thorax, and abdomen for staging as well as clinical review by senior members of the surgical service, gastroenterology, and general medicine. Following diagnostic work-up, patients attend a preoperative assessment clinic where they are examined by a senior member of the anesthetic and critical care service. Hematological and biochemical testing and, when indicated, electrocardiography and echocardiography are also completed. Results are reviewed and patients with treatable comorbidity are triaged for specific clinical care. Those with normal investigations, those with abnormal blood tests without overt clinical correlate (and so without ready correction), and those with conditions that cannot be further optimized but whom are judged suitable for operation all proceed to surgery.

### Data interpretation

The blood profiles of patients reviewed for this study were obtained within the time period after diagnosis and before the operation. All patients routinely have FBC measured while some have CRP and carcinoembryonic antigen (CEA) checked as part of their oncological staging and general work-up. The inflammatory markers included for analysis are CRP, white blood cell count (WBC), platelets, neutrophils, lymphocytes, platelets-to-lymphocyte ratio (PLR), and neutrophil-to-lymphocyte ratio (NLR). Hemoglobin (Hb) was also included as was CEA—although this tumor marker has a recognized role in monitoring colorectal cancer treatment and detecting recurrence, its role relative early postoperative morbidity and mortality has not been previously examined [20, 21].

Other patient-related factors recorded were age, gender, body mass index, and smoking status.

Preoperative comorbidities recorded include diabetes in addition to any defined cardiovascular, respiratory, renal and autoimmune disease known to preexist or detected on work-up. Cardiovascular comorbidities include ischemic heart disease, chronic heart failure, cardiomyopathies, murmurs, and atrial fibrillation. Respiratory comorbidities include asthma, chronic obstructive pulmonary disease, and interstitial lung disease. Renal comorbidities include chronic kidney disease, and autoimmune diseases include rheumatoid arthritis, Guillain-Barre syndrome, diabetes mellitus type I, and autoimmune thyroid disorders. Surgical technique was classified as laparoscopic or open, the latter including conversions to open. Tumor site was classified into right-sided (cecum, ascending, and transverse), left-sided (descending and sigmoid), and rectal. In patients with malignant tumors, pathological tumor stage, nodal status, and differentiation were recorded.

All 30-day postoperative outcomes and management were recorded prospectively for departmental audit. Various surgical site and systemic complications were recorded. Surgical site complications, both superficial and deep, include ileus, anastomotic leakage, and wound infection, dehiscence, or herniation. Systemic complications include thromboembolism, urinary, renal, respiratory, cardiovascular, and neurological complications. Urinary complications recorded include urinary incontinence and urinary tract infection. Renal complications include acute kidney injury. Respiratory complications include pneumonia and respiratory failure. Cardiovascular complications include new-onset arrhythmias, myocardial infarction, and decompensation of heart failure. Neurological complications include delirium.

Each patient was also assigned a Clavien-Dindo (CD) grade, which signifies postoperative complication severity. Under the CD classification, grade I signifies any deviation from the normal course that neither requires pharmacological treatment, with certain exceptions, nor endoscopic or radiological intervention. Grade II represents complications that require pharmacological treatment. Grade III represents complications that require other intervention. Grade IV is a life-threatening complication. Grade V represents death of the patient [22]. Patients with a normal postoperative course and no complications are not assigned a CD score.

### Statistical techniques

Continuous variables were presented as means with their standard deviations (***σ***) in tabular form. Preoperative and intraoperative values were transformed into categorical variables. For example, inflammatory markers were classified as normal or abnormal according to standard laboratory values. Though we used a cut-off of above 5 mg/L to denote CRP elevation, we also ran regression analysis using a cut-off of 10 mg/L to assess the validity of this threshold as used in the mGPS scoring system. Univariable analysis was performed using chi square analysis or Fisher’s exact test where appropriate, with these variables examined against all listed postoperative complications and Clavien-Dindo grades (Table 1). Multivariable analysis included only variables scoring *p* ≤ 0.25 in the univariable analysis and was performed using binary logistic regression analysis and expressed as odds ratios (OR). A ROC curve was plotted of CRP against (a) the presence and (b) the severity of a postoperative complication. Optimal cut-off values of CRP were determined using the Youden index. All analyses were performed using SPSS (SPSS, Version 20. Armonk, NY). Results were considered statistically significant where the two-tailed *p* value was less than 0.05.

**Table 1:** Univariable analysis for postoperative complications—OR (*p* value)

	Ileus	Wound infection/dehiscence/hernia	Anastomotic leak	Sepsis	Embolism	Renal	Resp	Cardio	Neuro	Clavien Dindo > 0	Clavien-Dindo 345
Age ≥ 65	0.8 (0.640)	0.7 (0.608)	1.0 (0.968)	1.2 (0.819)	0.5 (0.284)	2.2 (0.220)	2.8 (0.060)	3.1 (0.060)	4.0 (0.161)	1.8 (0.048)	1.6 (0.215)
Male sex	1.0 (0.948)	1.4 (0.611)	1.7 (0.054)	3.7 (0.195)	0.6 (0.461)	1.4 (0.585)	1.6 (0.292)	1.2 (0.670)	2.2 (0.330)	1.3 (0.364)	1.8 (0.221)
BMI ≥ 30	0.9 (0.882)	1.2 (0.781)	0.7 (0.145)	5.2 (0.040)	1.9 (0.419)	1.2 (0.746)	0.7 (0.559)	0.5 (0.333)	2.5 (0.255)	1.0 (0.928)	1.6 (0.255)
Smoker	1.2 (0.862)	1.5 (0.622)	1.3 (0.821)	1.0 (0.981)	2.1 (0.370)	3.7 (0.25)	3.4 (0.010)	2.4 (0.125)	1.0 (0.981)	1.8 (0.164)	2.3 (0.060)
Cardiovascular	2.1 (0.184)	1.0 (0.990)	1.2 (0.849)	1.8 (0.443)	0.8 (0.762)	0.8 (0.662)	2.0 (0.099)	3.6 (0.004)	1.2 (0.814)	1.3 (0.359)	1.1 (0.769)
Respiratory	1.2 (0.789)	1.0 (0.988)	0.8 (0.828)	0.7 (0.693)	1.3 (0.729)	0.9 (0.882)	1.3 (0.613)	0.2 (0.064)	0.5 (0.489)	1.2 (0.636)	0.4 (0.065)
Renal	0.7 (0.685)	1.0 (0.982)	2.1 (0.396)	0.7 (0.713)	2.6 (0.198)	2.7 (0.063)	1.7 (0.269)	2.8 (0.026)	2.1 (0.295)	1.8 (0.096)	1.7 (0.205)
Diabetes	(0.368)	(0.999)	1.1 (0.942)	0.9 (0.923)	1.9 (0.455)	3.7 (0.012)	1.4 (0.519)	1.3 (0.647)	2.9 (0.134)	0.9 (0.797)	1.1 (0.772)
Autoimmune	1.1 (0.901)	0.7 (0.696)	3.2 (0.174)	2.5 (0.269)	2.1 (0.374)	1.4 (0.590)	2.7 (0.036)	1.5 (0.505)	0.7 (0.785)	2.5 (0.025)	2.9 (0.010)
Malignant tumors	1.2 (0.849)	0.9 (0.926)	0.5 (0.519)	(0.999)	(0.999)	1.6 (0.674)	0.5 (0.209)	1.0 (0.953)	0.9 (0.330)	1.2 (0.702)	2.2 (0.290)
Rectal tumous	0.6 (0.528)	0.4 (0.333)	0.8 (0.181)	(0.354)	1.2 (0.862)	1.1 (0.802)	0.8 (0.753)	2.3 (0.071)	(0.214)	0.6 (0.101)	0.4 (0.080)
Open approach*	2.8 (0.070)	1.0 (0.952)	2.1 (0.376)	3.3 (0.108)	0.6 (0.621)	3.7 (0.010)	2.7 (0.024)	1.8 (0.255)	3.6 (0.050)	3.2 (0.001)	2.0 (0.072)
Hemoglobin < 10 g/dl	2.8 (0.093)	0.6 (0.665)	1.2 (0.893)	4.7 (0.033)	3.7 (0.064)	0.9 (0.873)	0.8 (0.742)	0.6 (0.478)	3.1 (0.108)	2.3 (0.040)	1.6 (0.299)
WCC < 4× 10^9^/L	1.0 (0.721)	1.0 (0.755)	(0.999)	(0.999)	(0.999)	(0.999)	(0.999)	(0.999)	(0.999)	(0.498)	(0.999)
WCC > 11× 10^9^/L	1.3 (0.786)	(0.999)	(0.999)	2.8 (0.345)	(0.999)	4.4 (0.025)	3.9 (0.024)	3.1 (0.090)	(0.999)	6.2 (0.008)	8.3 (0.000)
Platelet < 150× 10^9^/L	(0.999)	(0.999)	10.3 (0.018)	8.5 (0.032)	(0.999)	3.3 (0.277)	12.9 (0.001)	2.5 (0.402)	(0.999)	1.6 (0.621)	2.9 (0.225)
Platelet > 400× 10^9^/L	1.2 (0.855)	(0.999)	(0.999)	2.5 (0.394)	5.4 (0.030)	4.0 (0.038)	1.3 (0.743)	3.0 (0.104)	15.8 (0.000)	6.9 (0.005)	6.8 (0.000)
Lymphocytes < 1× 10^9^/L	3.1 (0.048)	2.0 (0.315)	2.3 (0.324)	1.9 (0.462)	0.6 (0.677)	0.6 (0.548)	1.5 (0.411)	1.5 (0.471)	1.3 (0.740)	1.4 (0.343)	1.9 (0.105)
Neutrophils < 2× 10^9^/L	1.0 (0.719)	(0.999)	(0.999)	(0.999)	(0.999)	(0.999)	(0.999)	(0.999)	(0.999)	(0.493)	(0.999)
Neutrophils > 8× 10^9^/L	1.1 (0.913)	(0.999)	(0.999)	2.3 (0.437)	2.0 (0.529)	5.7 (0.003)	4.5 (0.006)	1.5 (0.625)	1.7 (0.615)	7.4 (0.003)	5.8 (0.000)
PLR < 61	1.0 (0.799)	(0.999)	(0.999)	(0.999)	(0.999)	(0.999)	(0.999)	(0.999)	(0.999)	(0.999)	(0.999)
PLR > 239	2.3 (0.146)	1.1 (0.915)	1.3 (0.790)	1.0 (0.997)	2.6 (0.170)	1.6 (0.800)	0.7 (0.595)	0.8 (0.715)	3.3 (0.066)	1.7 (0.080)	2.1 (0.041)
NLR < 0.83	(0.999)	(0.999)	(0.999)	(0.999)	(0.999)	(0.999)	(0.999)	(0.999)	(0.999)	(0.999)	(0.999)
NLR > 3.92	1.5 (0.523)	2.4 (0.172)	4.8 (0.051)	0.9 (0.908)	1.4 (0.664)	3.2 (0.020)	2.3 (0.044)	1.8 (0.198)	3.0 (0.096)	3.4 (0.000)	3.9 (0.000)
CRP ≥ 5 mg/L	2.2 (0.356)	1.6 (0.593)	5.7 (0.079)	(0.052)	(0.024)	15.4 (0.001)	4.5 (0.007)	7.6 (0.003)	(0.052)	16.9 (0.000)	20.5 (0.000)
CRP ≥ 10 mg/L	2.4 (0.281)	1.0 (0.008)	10.1 (0.014)	1.1 (0.002)	10.1 (0.014)	16.9 (0.001)	10.4 (0.000)	3.6 (0.021)	1.1 (0.002)	106.3(0.000)	63.3 (0.000)
CEA ≥ 3 ng/mL	1.1 (1.000)	0.2 (0.123)	0.3 (0.373)	1.1 (0.872)	1.5 (0.575)	1.6 (0.406)	1.0 (0.935)	1.5 (0.425)	3.0 (0.254)	1.9 (0.036)	1.3 (0.551)
T stage ≥ 4	2.3 (0.213)	(0.999)	4.7 (0.073)	1.1 (0.931)	(0.600)	1.0 (0.987)	1.1 (0.876)	2.7 (0.076)	2.0 (0.413)	2.6 (0.033)	1.6 (0.328)
Node positive	0.3 (0.123)	2.2 (0.254)	2.6 (0.294)	2.3 (0.276)	1.0 (0.997)	0.8 (0.725)	0.8 (0.672)	1.2 (0.667)	2.2 (0.254)	0.9 (0.594)	1.0 (0.897)
Moderate/poorly differentiated	3.4 (0.220)	0.6 (0.437)	0.4 (0.351)	1.8 (0.587)	0.9 (0.876)	0.8 (0.705)	1.3 (0.670)	0.4 (0.034)	0.6 (0.437)	1.0 (0.926)	0.8 (0.628)

Results expressed as odds ratios (OR) with p values in brackets. Variables with *p* < 0.25 in this table are included for multivariable analysis. Where there were null values in contingency tables for univariate analysis, OR was not calculated and Fisher’s exact test used for *p* value and insertion to multivariate analysis where < 0.25. *versus laparoscopic

## Results

During the time period, 198 patients underwent surgical resection for colorectal cancer and 20 likewise for colorectal dysplasia (total *n* = 218). Over 80% of these operations were performed laparoscopically. Patient demographics and tumor characteristics are shown in Table 2, with most cancers located on the left-side, the majority being node-negative and moderately differentiated. Complete data was available on all patients regarding operation type and approach, tumor characteristics, and postoperative complications. All patients underwent preoperative hematological testing. However, only a proportion had CRP (63.8%, *n* = 139) and CEA (78.4%, *n* = 171) measured prior to surgery. Between the patients who underwent preoperative CRP testing and those who did not, there was no difference in pre-existing comorbidities (Pearson coefficient *r* = − 0.008, *p* = 0.906) or in postoperative complications (Phi coefficient φ = 0.112, *p* = 0.099). Almost half of patients had a postoperative complication, with 18.8% (*n* = 41) graded as a Clavien-Dindo grade ≥ 3, requiring surgical intervention (Table 2). Mean preoperative laboratory values are also reported in Table 2. Mean CRP in this group of patients presenting for elective colorectal resection was 18.7 mg/L (σ 34.1), with 48.3% having a CRP ≥ 5 mg/L and 30.2% ≥ 10 mg/L.

**Table 2:** Patient data

Patient demographics		Tumor data		Preoperative laboratory values		Postoperative complications		Clavien-Dindo grades	
**Variable**	***n*** **(%)**	**Variable**	***n*** **(%)**	**Variable**	***x̅*** ***[s]***	**Variable**	***n*** **(%)**	**Variable**	***n*** **(%)**
Age > 65	147 (67.4)	**Tumor site**		Hb (g/dL)	12.4 [2.1]	Ileus	13 (6.0)	No complications	111 (50.9)
Male	136 (62.4)	Rectum	49 (22.5)	WBC (× 10^9^/L)	7.4 [2.2]	Wound infection	8 (3.7)	CD I	52 (23.9)
BMI ≥ 30	43 (29.9)	Left colon	90 (41.3)	Platelets (× 10^9^/L)	273 [82.6]	Wound dehiscence	3 (1.4)	CD II	14 (6.4)
**Patient comorbidity**		Right colon	79 (36.2)	Neutrophils (× 10^9^/L)	4.8 [1.9]	Hernia	7 (3.2)	CD III	12 (5.5)
Cardiovascular	65 (29.8)	Tumor stage T3/T4	120 (60.9)	Lymphocytes (× 10^9^/L)	1.5 [0.6]	Anastomotic leak	6 (2.8)	CD IV	27 (12.4)
Respiratory	44 (20.2)	Node-positive	74 (37.6)	PLR	208 [125.2]	Sepsis	7 (3.2)	CD V	2 (0.9)
Renal	43 (19.7)	Malignant	198 (90.8)	NLR	4.0 [4.5]	Embolism	8 (3.7)		
Diabetes	34 (15.6)	Moderately/poorly differentiated	192 (97)	CRP (mg/L)	18.7 [34.1]	Urinary	16 (7.3)		
Autoimmune	31 (14.2)			CEA (ng/mL)	8.4 [24.7]	Renal	16 (7.3)		
Smoker	30 (16.3)					Respiratory	25 (11.5)		
Laparoscopic	176 (80.7)					Cardiovascular	21 (9.6)		
						Neurological	9 (4.1)		

*x̅* mean, *s* standard deviation, *Hb* hemoglobin, *WBC* white blood cells, *PLR* platelet to lymphocyte ratio, *NLR* neutrophil to lymphocyte ratio, *CRP* C-reactive protein, *CEA* carcinoembryonic antigen, *CD* Clavien-Dindo

Predictors of postoperative morbidity were analyzed using logistic regression analysis, with elevated preoperative CRP being the strongest factor to consistently achieve significance in multivariable analysis for morbidity (Tables 3 and 4). Preoperative CRP ≥ 5 mg/L was the only factor significantly predictive of all-cause mortality, with an OR of 17.0 (*p* < 0.001) and was the strongest factor to predict a Clavien-Dindo morbidity grade ≥ 3 (OR 41.9, *p* < 0.001). Other factors predictive of Clavien-Dindo morbidity grade ≥ 3 included smoking, elevated preoperative platelet count, and elevated preoperative neutrophil-lymphocyte ratio (OR 15.6, 8.6, and 6.3, respectively, all *p* < 0.05).

**Table 3:** Multivariable analysis for postoperative complications with CRP cut-off ≥ 5 mg/L

	Ileus	Sepsis	Embolism	Renal	Resp	Cardio	Neuro	Clavien–Dindo > 0	Clavien–Dindo 345
Smoker				15.7 (0.003)	5.6 (0.016)				15.6 (0.008)
Cardiovascular						13.9 (0.05)			s
Respiratory									0.1 (0.005)
Renal						18.0 (0.032)			
Rectal tumors						60.4 (0.017)			
Open approach		11.5 (0.05)							
Platelet > 400 × 10^9^/L			14.1 (0.014)				54.4 (0.001)		8.6 (0.035)
Lymphocytes < 1 × 10^9^/L	0.3 (0.030)								
Neutrophils > 8 × 10^9^/L					7.0 (0.031)				
NLR > 3.92									6.3 (0.010)
CRP ≥ 5 mg/L				17.0 (0.013)	10.5 (0.007)			17.0 (0.000)	41.9 (0.001)

Results expressed as odds ratios (OR) with *p* values in brackets. Only significant results (*p* ≤ 0.05) are included here

**Table 4:** Multivariable analysis for postoperative complications with CRP cut-off ≥ 10 mg/L

	Embolism	Renal	Resp	Cardio	Neuro	Clavien–Dindo > 0	Clavien–Dindo 345
Age ≥ 65						5.0 (0.049)	
Smoker		5.2 (0.030)					209.5 (0.013)
Cardiovascular				34.3 (0.005)			
Respiratory							0.0 (0.000)
Renal							28.8 (0.033)
Malignant tumors			0.1 (0.038)				
Rectal tumors				32.0 (0.022)			
Open approach							21.3 (0.039)
Plt > 400	13.8 (0.015)				24.8 (0.012)		
NLR > 3.92							7.7 (0.041)
CRP ≥ 10 mg/L		13.1 (0.002)	36.7 (0.001)	59.6 (0.010)			2762.6 (0.001)
Moderate/poorly differentiated				0.1 (0.029)			

Results expressed as odds ratios (OR) with p values in brackets. Only significant results (*p* ≤ 0.05) are included here

In particular, both patients with an elevated preoperative CRP and patients who smoke were likely to have renal (OR 17 and 15.7, respectively, both *p* < 0.05) and respiratory (OR 10.5 and 5.6 respectively, both *p* < 0.05) postoperative complications. An elevated neutrophil count was associated with postoperative respiratory complications (OR 7, *p* < 0.05). Unsurprisingly, an open surgical approach was linked to postoperative sepsis (OR 11.5, *p* = 0.05), and an elevated preoperative platelets count was linked to postoperative thromboembolism as well as neurological complications (OR 14.1 and 54.4 respectively, both *p* < 0.05).

**Fig. 1:**
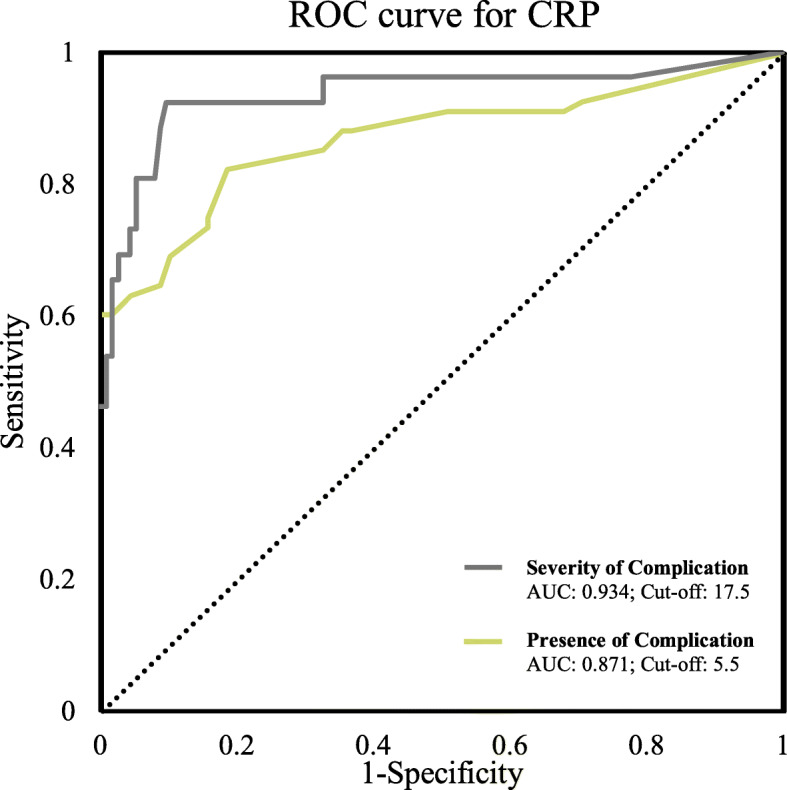
Receiver operator characteristic curves for CRP in association with the presence of any and severe complications by CD grade. AUC: area under curve

When the CRP cut-off of 10 mg/L was incorporated into the analysis, CRP remained the preoperative factor most predictive for morbidity with the highest ORs among all inputted variables. However, CRP lost significance for the ability to predict all-cause morbidity. All other examined factors failed to consistently predict postoperative morbidity. ROC curve calculations indicated the diagnostic ability of CRP in the prediction of any postoperative complication (CD 1–5) and severe complications (CD 3–5). Area under the curve (AUC) is 0.871 and 0.934 for any and severe complications, respectively. Youden index cut-off values of CRP after which a patient is more likely to have any complication was 5.5 mg/L and the value after which a patient is more likely to have a severe postoperative complication, i.e., CD ≥ 3, was 17.5 mg/L (Fig. 1).

## Discussion

Routine preoperative and postoperative care in major elective colorectal surgery includes measurement of blood tests for the identification of baseline and subsequent trends in physiological derangement induced by surgery. In this way, minor and major complications can be diagnosed early, arousing suspicion before clinical symptoms. On this account, prompt management can minimize adverse outcomes. Standard hematological markers such as WCC or neutrophils are relatively weak prognosticators in this regard and hence additional biomarker screening should be performed including CRP [7]. CRP is an acute phase protein with a short half-life (19 h), synthesized by the liver in a non-specific response to a variety of provocations mediated via proinflammatory cytokines, such as interleukin-6. Its levels rise in response to trauma, infection, ischemia, and malignancy.

Although not commonly recommended in guidelines related to preoperative work-up [8], CRP is nonetheless quite commonly ordered as part of oncological staging, and elevated levels in patients without obvious acute malady presenting for and proceeding with elective surgery have been previously reported. Irrespective of this, in most clinical settings only the postoperative CRP levels are followed. Recent clinical research monitors postoperative systemic inflammation, quantified by the mGPS of which CRP and albumin are constituents. Various systemic reviews and meta analyses have shown that postoperative mGPS has a strong prognostic value in the overall survival and recurrence-free survival of patients with colorectal cancer [23–25]. While effort is directed towards following systemic inflammation postoperatively, one particular study by Moyes et al. correlates preoperative mGPS and white cell count to postoperative complications in patients undergoing curative resection for colorectal cancer, as shown in Tables 1 and 2 in their paper [26].

Systemic inflammation, besides concomitant sepsis and inflammatory pathology, is independently associated with cancer. Standard practice has been to out rule gross sepsis prior to surgery, through clinical evaluation and baseline FBC. The absence of overt infection on preoperative blood tests may formerly have reassured. However, the results herein suggest that even a subtle rise in preoperative CRP may warrant stratifying patients into a higher risk group. Here, we have looked more broadly across the spectrum of morbidity encountered and found correlations worthy of future prospective study. The strong association between preoperative CRP and CD grade ≥ 3 morbidity may indicate its use as a clinical tool in the context of preoperative assessment.

Baseline inflammatory mediator levels therefore may predict complications, but yet they themselves can be moderated by preoperative prehabilitation programs [5]. Patients with cardiovascular disease are known to have elevated systemic inflammatory mediators that are responsive to exercise training.

Recent research show that patients presenting for surgery for colorectal cancer can significantly engage with and benefit from preoperative exercise even for short intervals [27, 28]. Improved postoperative outcomes have been demonstrated in some studies, pointing to a possible shared mechanism or marker of inflammatory mediators. Other interventions could include nutrition and microbiome ecology, even if specific anti-inflammatory pharmacology is avoided due to potential wound healing complications. Interestingly in the latter regard, recent work has linked single perioperative steroid usage as part of anesthetic induction with improved postoperative sequence [29].

The biggest limitation of this study is its retrospective nature, which serves a purpose only in exploring a hypothesis to help its evaluation ahead of a prospective study for proper elucidation and determination. It does not prove causative or mechanistic relationships. A number of patients in this study did not undergo preoperative CRP testing. Correlation analyses show that there is no difference in pre-existing comorbidities or in postoperative complications between patients who underwent CRP testing versus those who did not, suggesting that there was no clinically distinguishing overt condition which prompted clinicians to order CRP test for only some patients. Rather, this can be explained by that CRP is not as routine of a preoperative test to perform as is full blood count. We suggest a prospective study in order to confirm the significant findings yielded in this audit.

## Conclusion

This study demonstrates that elevated preoperative CRP ≥ 5 mg/L is a strong predictor of all-cause mortality, and CRP ≥ 17.5 mg/L predicts severe postoperative complications as depicted in Clavien-Dindo grades 3–5. Other strong predictors of severe postoperative complications are smoking, an elevated preoperative platelet count, and an elevated preoperative neutrophil-lymphocyte ratio. Our findings suggest that routinely measuring CRP of patients at the time of preoperative assessment may notably assist risk stratification. The concept of an inflammatory continuum bridging preoperative and postoperative timeframes should be built into prospective studies to confirm this finding and explain causative, downstream effectors.

## Data Availability

The datasets generated and analyzed in our study are available from the first author on reasonable request.
